# Research on the Three Categories of Oxygen-Barrier Packaging Materials for the Effect of Chilled Chicken Preservation

**DOI:** 10.3390/foods15162897

**Published:** 2026-08-19

**Authors:** Debao Wang, Xiaoyu Chai, Su Wang, Yanan Zang, Weili Rao, Xin Li, Dequan Zhang, Chengli Hou

**Affiliations:** 1 Institute of Food Science and Technology, Chinese Academy of Agricultural Sciences, Key Laboratory of Agro-Products Quality and Safety in Harvest, Storage, Transportation, Management and Control, Ministry of Agriculture and Rural Affairs, Beijing 100193, China; wangdebao@caas.cn (D.W.); 15027708107@163.com (X.C.); wangsu0630@163.com (S.W.); zangyanan_zyn@163.com (Y.Z.); lixin@caas.cn (X.L.); dequan_zhang0118@163.com (D.Z.); 2 College of Food Science and Technology, Hebei Agricultural University, Baoding 071000, China; 15931808752@163.com

**Keywords:** chicken, barrier packaging, dominant spoilage bacteria, freshness, shelf life

## Abstract

Determining the oxygen barrier packaging material is crucial for maintaining the quality and freshness of chilled chicken during low-temperature storage. Chilled chicken was packaged in three materials with different oxygen permeability: medium-oxygen-barrier packaging (MORP, 23.95 cm^3^/(m^2^·24 h·0.1 MPa)), low-oxygen-barrier packaging (LORP, 1631.44 cm^3^/(m^2^·24 h·0.1 MPa)), and polyethylene packaging (OPP, 10,600.00 cm^3^/(m^2^·24 h·0.1 MPa)). The packaged samples were then stored at 4 °C for 9 d. Compared to the OPP group, where *Pseudomonas*, *Acinetobacter*, and *Lactobacillus* dominated, MORP and LORP significantly inhibited *Pseudomonas* growth, resulting in slower microbial population growth during storage. Owing to the distinct composition of dominant spoilage bacteria, the LORP group demonstrated significantly lower levels of sulfide and nitrogen oxides during storage compared to other groups. Within the first 7 days, the LORP group exhibited markedly lower TVB-N levels in comparison to both the MORP and OPP groups. During the late storage stage, the muscle fiber structure in both the MORP and LORP groups remained relatively intact. In summary, packaging materials with low to medium barrier properties are more effective in maintaining chicken freshness and extending its shelf life to up to 9 days. This method potentially provides an effective foundation for maintaining the quality of poultry meat.

## 1. Introduction

Chicken is a nutrient-dense food, rich in high-quality protein and low in fat, and its amino acid composition closely aligns with human nutritional requirements [1]. With the global population increasing and consumer demand for high-protein, low-fat poultry products rising, the global chicken production sector has witnessed sustained growth [2]. Currently, the barrier properties of packaging materials exhibit a diverse range of characteristics [3,4]. Selecting packaging that meets chicken freshness requirements has become a primary consideration [5,6]. If the barrier properties of packaging materials are inadequate for chicken preservation requirements, it would lead to ineffective microbial inhibition, insufficient shelf-life extension, and consequently increased food waste and higher costs [7]. Therefore, it is crucial to identify an effective barrier packaging material that is appropriate for preserving the quality and safety of chilled chicken.

The barrier performance of packaging materials is the paramount factor in extending the shelf life of meat and maintaining its quality over an extended period [5,6,7]. Depending on their barrier properties, these materials can be classified into three categories: high-barrier, medium-barrier, and low-barrier/breathable materials [8,9]. Oxygen permeability is a critical parameter that significantly influences meat quality [10]. Many microorganisms, including species from the genera Bacillus and *Pseudomonas*, are obligate aerobes that rely on oxygen for aerobic respiration [11]. The pivotal role of oxygen as the terminal electron acceptor in oxidative phosphorylation underpins efficient energy production, which ultimately sustains cellular biosynthesis and division [12,13]. Insufficient oxygen levels can severely impair or completely arrest the growth and metabolic activities of these microorganisms [12,13]. Simultaneously, meat contains a variety of endogenous enzymes, including lipoxygenase and polyphenol oxidase [14]. In the presence of oxygen, these enzymes catalyze the oxidation of specific substrates, thereby accelerating meat degradation. Kartika et al. [15] demonstrated that vacuum packaging of chicken legs with varying oxygen permeability levels (<10, 3000, and ≥7000 cm^3^/(m^2^·24 h)) significantly reduced microbial counts in the product. Their findings demonstrated that enhancing the barrier properties of these packaging materials improved the quality retention and extended the shelf life of the product.

In contrast to myoglobin-rich lamb and beef, chicken contains lower levels of myoglobin [16]. Consequently, it typically exhibits a pale pink or white coloration and does not undergo significant color changes upon oxygen exposure. The fat content of chicken is relatively small, and its fatty acid composition is also different from that of beef and lamb, especially the unsaturated fatty acid content is low [17]. Therefore, in an oxygen-rich environment, the oxidation rate of chicken fat is comparatively low, resulting in minimal changes in flavor, which indicates a lower sensitivity to oxygen. Medium-barrier packaging is widely employed for the preservation of chicken. This packaging method can maintain the quality of chicken for several days under refrigerated conditions, thereby satisfying short-term consumer needs [18]. However, the specific barrier properties required for optimal fresh chicken preservation have not yet been clearly established.

**Scheme 1:**
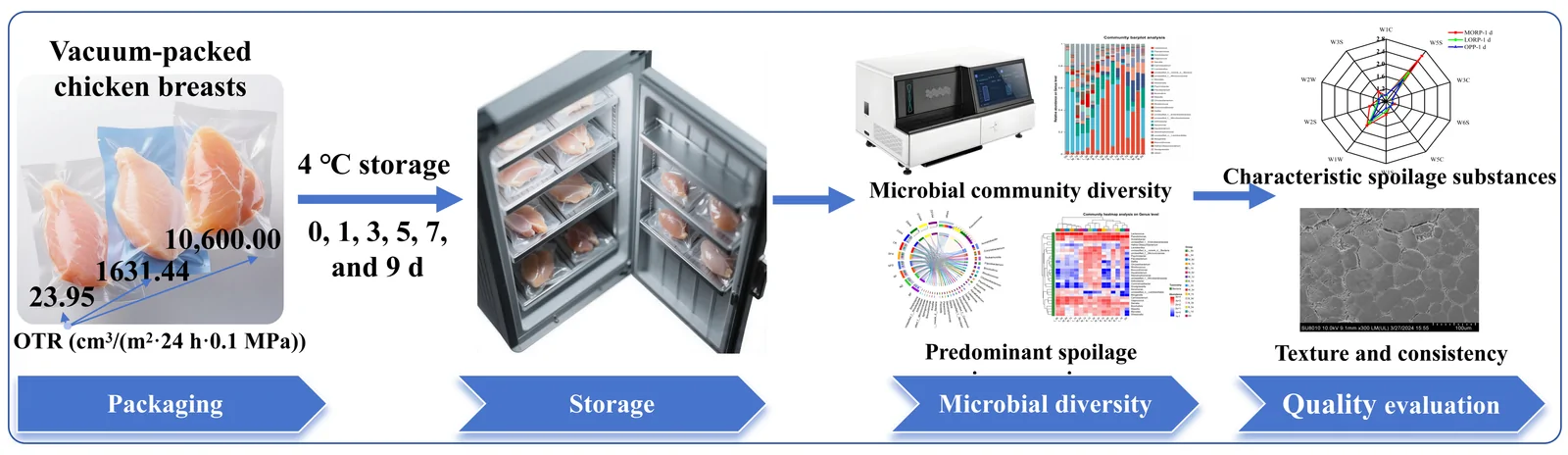
Effect of packaging material barrier properties on the quality of fresh chicken breast meat.

In this study, to ascertain the barrier requirements for fresh chicken preservation, three composite films—AHE (PA/EVOH/PE), AHE (PA/PE), and PPE (PP/PE)—with thicknesses of 150, 150, and 175 μm and oxygen transmission rates of 23.95, 1631.44, and 10,600.00 cm^3^/(m^2^·24 h·0.1 MPa), respectively, were evaluated. This assessment was based on a thorough evaluation of the microflora composition, total viable bacteria count, pH levels, color attributes, protein oxidation, sensory characteristics, and microstructural changes during storage at 4 °C. This study is expected to provide a robust theoretical foundation for selecting optimal packaging materials to maximize the preservation of fresh poultry (Scheme 1).

## 2. Materials and Methods

### 2.1. Materials

Fresh breast samples were procured from Hebei Meikeduo Food Group Co., Ltd. (Cangzhou, China). Packaging materials were supplied by Sunrise Material Co., Ltd. (Jiangyin, China). Plate count agar (pH 7.0 ± 0.2) was obtained from Landbridge Technology Co., Ltd. (Beijing, China). Hydrochloric acid (0.01 mol/L) and carbamide were sourced from Sinopharm Chemical Reagent Co., Ltd. (Shanghai, China). Trichloroacetic acid was purchased from Aladdin Biochemical Technology Co., Ltd. (Shanghai, China). 2,4-dinitrophenylhydrazine hydrochloric acid buffer solution was acquired from Qiyan Biotechnology Co., Ltd. (Beijing, China).

### 2.2. Experimental Design

The experiment consisted of three experimental groups, each utilizing different packaging materials: medium-oxygen-barrier packaging (MORP), low-oxygen-barrier packaging (LORP), and oxygen-permeable packaging (OPP). They are three types of packaging films co-extruded from composite materials—namely polyamide/ethylene-vinyl alcohol copolymer/polyethylene composite materials (AHE), polyamide/ polyethylene composite materials (AAE), and polypropylene/polyethylene composite materials (PPE). The oxygen barrier rates for these materials were 23.95 cm^3^/(m^2^·24 h·0.1 MPa), 1631.44 cm^3^/(m^2^·24 h·0.1 MPa), and 10,600.00 cm^3^/(m^2^·24 h·0.1 MPa), respectively. The thicknesses of the films are 150, 150, and 175 μm, respectively. All samples were vacuum-packed using vacuum packaging machines and then stored at 4 °C. The three experimental groups were as follows: whole chicken breast on the left were used and packed with MORP, LORP, and OPP materials. Samples were collected at intervals of 0, 1, 3, 5, 7, and 9 d to analyze for total viable count, pH, color, protein oxidation, and microstructure. The biological replicate for each sample is *n* = 6. The remaining samples were stored at −80 °C for subsequent determination of volatile basic nitrogen (TVB-N) and analysis of volatile odor changes using an electronic nose.

### 2.3. Microbial Community Characterization

The bacterial solution from different meat samples was centrifuged at 4 °C for 10 min at 2000 rpm. The supernatant was then removed, and the sediment was subjected to a second centrifugation at 4 °C for 12,000 rpm. The final sediment was stored at −80 °C. The V3-V4 region of the bacteria was amplified using Illumina Miseq sequencing with primers 338F (5′-ACTCctacgggaggcagcagg-3′) and 806R (5′-GGACTACNNGGGTATCTAAT-3′). Sequencing results were used to assign operational taxonomic units (OTUs) based on 97% similarity for species identification. OTU and Silva databases were utilized to measure species diversity. Alpha diversity indices (Chao1, Observed_species, PD_whole_tree, Shannon) were calculated, and UniFrac analysis was performed to assess microbiota composition differences and Beta diversity.

### 2.4. Total Viable Count (TVC)

An accurately weighed meat sample of 10.00 g was transferred into 90 mL of sterile saline solution in accordance with the determination method specified in GB 4789.2-2022 [19]. The sample was subjected to uniform agitation for 5 min, after which 0.5 mL was withdrawn and added to 4.5 mL of sterile saline to achieve a tenfold dilution. Following dilution, three appropriate gradients were selected, each comprising three parallel samples. A total volume of 100 μL from each gradient was aspirated and spread onto plates, then incubated at 37 °C for 24 h.

### 2.5. pH Value and Color

The pH value of each meat sample was measured using a handheld portable pH meter (Testo 205, Testo, Lenzkirch, Germany). The color of the meat samples was determined at 25 °C immediately after opening the packaging using a D65 colorimeter (CM-600d, Konica Minolta, Tokyo, Japan). Prior to measurement, the colorimeter was calibrated according to the manufacturer’s instructions. The L*, a*, and b* color values of the meat samples were measured six time at different locations and recorded.

$$\Delta E^{*}=\sqrt{{\left(L_{n}^{*}-L_{0}^{*}\right)}^{2}+{\left(a_{n}^{*}-a_{0}^{*}\right)}^{2}+{\left(b_{n}^{*}-b_{0}^{*}\right)}^{2}}$$

where $L_{n}^{*}$ represents the L* value for days 0 to 9, and n represents days 0 to 9. $a_{n}^{*}$ and $b_{n}^{*}$ are same with $L_{n}^{*}$. $L_{0}^{*}$, $a_{0}^{*}$, and $b_{0}^{*}$ are the L*, a*, and b* color values of the meat samples at day 0.

### 2.6. Total Sulfhydryl Content

A total of 1.00 g of breast was homogenized with 10 mL of 2% SDS buffer at 10,000 rpm for three 30 s intervals. The mixture was then centrifuged at 4 °C and 4000× *g* for 20 min to obtain the supernatant. Protein concentration was adjusted to 2.00 mg/mL using a BCA protein kit (Thermo Fisher Scientific Inc., Waltham, MA, USA). Next, 0.50 mL of the protein solution was mixed with 2.50 mL Tris-Gly-8 M urea buffer and 0.02 mL of 4.00 mg/mL DTNB solution. This mixture was incubated at 25 °C for 30 min, and OD412 was measured using the multifunctional microplate reader (FlashSpec SuperMax 3000FA, Shanghai Flash Spectrum Biotechnology Co., Ltd., Shanghai, China). Total sulfhydryl content (−SH μmol/g protein) was calculated using the provided formula.

(1) $$-SH(\mu \mathrm{mol}/g\;\mathrm{Pro})=\frac{75.53\times OD_{412}\times a}{b}$$

where a (6.04) is the dilution coefficient, and b is the protein concentration in the sample.

### 2.7. Carbonyl Content

A total of 200 μL of protein solution (2.0 mg/mL) was added to a 10% TCA solution, then centrifuged at 12,000 rpm for 5 min at 4 °C. The supernatant was discarded, and the mixture was vortexed with 2,4-dinitrophenylhydrazine for a 30-min dark reaction. After re-centrifugation, the supernatant was decanted, and TCA solution was added. An ethanol-ethyl acetate solution was used to precipitate the mixture. Insoluble substances were removed with phosphate buffer, and absorbance was measured at 280 nm and 370 nm after 15 min using the multifunctional microplate reader (FlashSpec SuperMax 3000FA, Shanghai, China).

(2) $$\mathrm{Carbonyl}\;\mathrm{content}(\mathrm{nmol}/\mathrm{mg}\;\mathrm{Pro})=\frac{OD_{370}}{22000\times (OD_{280}-OD_{370}\times 0.43)}\times 10^{6}$$

### 2.8. Volatile Odor

The chicken breast sample, weighing exactly 2.00 g, should be transferred to a designated flavor bottle. The volatile odor of fresh chicken was measured using the electronic nose developed by Airsense Analytics (PEN 3.5, Mecklenburg, Germany). The sensor array response should be analyzed, with data from 48 to 52 s used for further analysis. Proteins and lipids serve as the principal chemical constituents of chilled chicken meat. During refrigerated storage, oxidative degradation of these components generates characteristic spoilage volatiles, including ammonia, amines, organic acids, and sulfur-containing compounds (Table 1).

**Table 1:** Electronic nose sensor arrays and their performance characteristics.

Sensor Serial Number	Sensor Model	Highly Sensitive Gas Type
1	W1C	Sensitive to aromatic hydrocarbons
2	W5S	Sensitive to nitrogen oxides
3	W3C	Sensitive to ammonia and aromatic compounds
4	W6S	Sensitive to hydrides
5	W5C	Sensitive to short-chain alkanes and aromatic compounds
6	W1S	Sensitive to methyl-containing compounds/Sensitive to methyl groups
7	W1W	Sensitive to sulfides
8	W2S	Sensitive to alcohols and aldehydes/ketones
9	W2W	Sensitive to organic sulfides and aromatic compounds
10	W3S	Sensitive to long-chain alkanes

### 2.9. Total Volatile Base Nitrogen (TVB-N)

The TVB-N content of the meat sample was determined in accordance with GB 5009.228-2016 [20]. A precisely weighed 10.00 g meat sample was homogenized for 20 s in 50 mL of 20 g/L trichloroacetic acid, followed by incubation on ice for 30 min and subsequent filtration. A total of 10 mL of the filtrate with 1.0 g magnesium oxide was transferred to a protein digestion tube connected to a Kjeldahl nitrogen analyzer (FOSS Company, Hillerød, Danish). During distillation, 10 mL of a 20 g/L boric acid solution was added, with a distillation duration of 5 min and 10 mL of leaching water. Finally, the distilled liquid, after mixing with 5 drops of indicator, was titrated using 0.01 M HCl, and the volume was recorded.

### 2.10. Chicken Breast Muscle Fiber Morphology

Samples were quickly frozen in liquid nitrogen, fixed in 2.5% glutaraldehyde, rinsed 2–3 times with 0.1 M PBS, and then washed with distilled water. They were dehydrated using ethanol of varying concentrations at 25 °C for 2 h. After gold spraying, muscle fiber relaxation was observed and captured using SEM (SU8010, Hitachi, Hitachinaka, Ibaraki, Japan).

### 2.11. Statistical Analysis

Statistical analysis was conducted using SPSS 27.0, with one-way analysis of variance (ANOVA) and the least-squares method used to assess the impact of packaging barrier and storage temperature on variables at *p* < 0.05. Figures were created using Origin 21.

## 3. Results

### 3.1. Effects of Packaging Barrier on Microbial Diversity in Chilled Chicken During Storage

**Figure 1:**
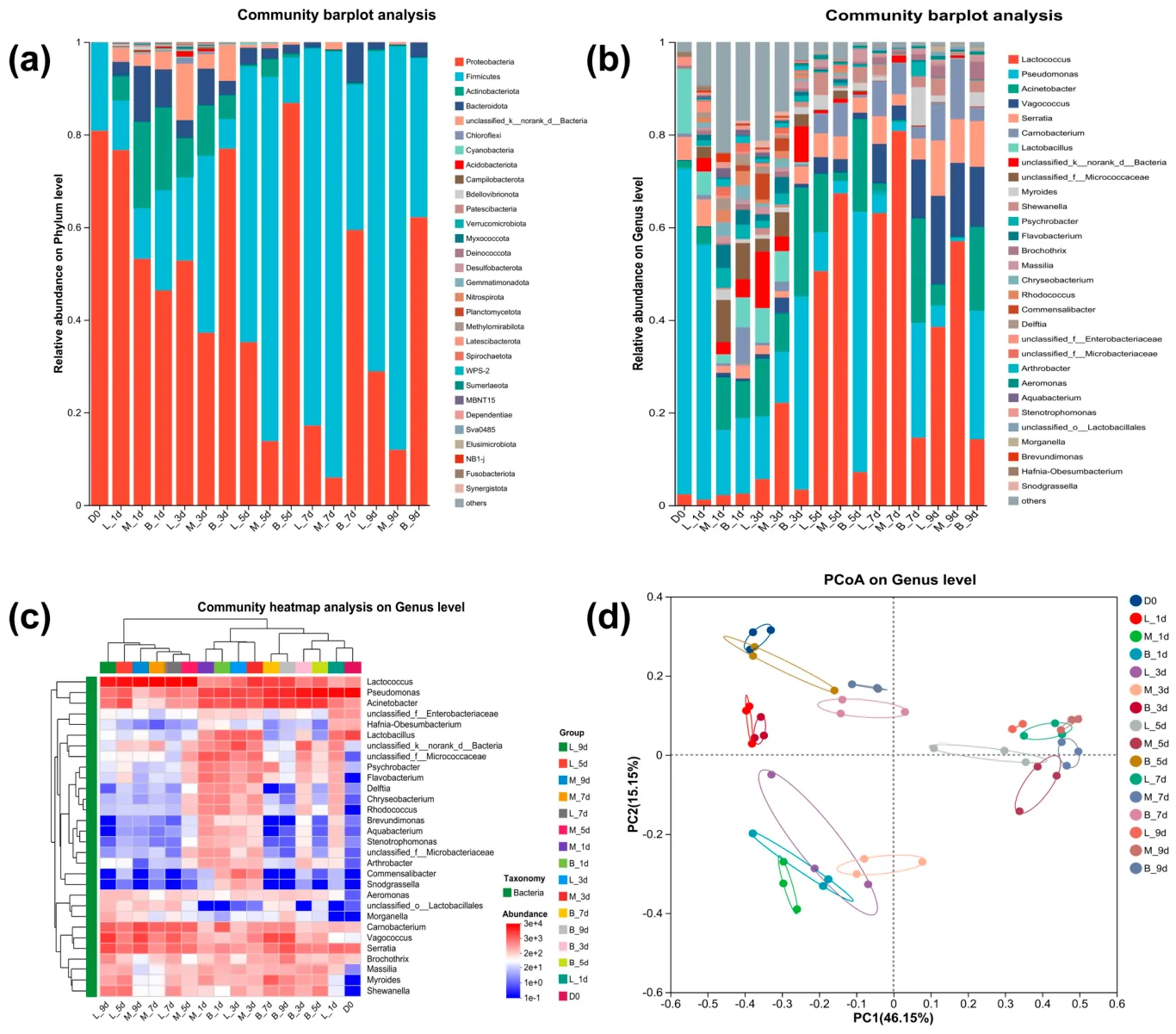
Changes in the rule of bacterial flora of chilled chicken under various barrier packaging. (**a**) Community abundance at the phylum level; (**b**) community abundance at the genus level; (**c**) community heatmap at the genus level; (**d**) PCoA at the genus level. In (**c**,**d**), D0 indicates the samples at 0 days. The group designations M, L, and B correspond to the MORP, LORP, and OPP groups, respectively, followed by “nd” indicating the storage time.

The microbial community structure and diversity in chicken meat are closely related to its freshness. Clarifying the composition of the microbial flora in fresh meat during storage is crucial for selecting appropriate barrier packaging materials and storage temperatures to achieve effective antibacterial preservation. In this study, the microbial community compositions of chilled chicken packaged with three types of barrier materials during storage are shown in Figure 1. The dominant spoilage bacteria in chilled chicken during storage were primarily *Proteobacteria*, followed by *Firmicutes*. This finding was similar to the microbial community structure observed in lamb meat during storage [21]. *Pseudomonas* was the predominant microorganism on chicken surfaces at day 0 (Figure 1a). *Lactobacillus* gradually outpaced *Pseudomonas*, emerging as the dominant bacterial species as storage time increased. Throughout this process, *Lactobacillus*, *Pseudomonas*, *Carnobacterium*, and *Brochothrix* were the predominant bacterial genera, constituting the main microbial flora. These findings are consistent with the results reported by Cauchie et al. [22] and Lauritsen et al. [23]. The OPP group exhibited the highest abundance of Pseudomonas compared to the MORP and LORP groups, as shown in Figure 1b–d. In contrast, the groups with higher barrier properties showed significantly greater abundance of Lactobacillus than Pseudomonas. Studies by Sung et al. [24] and Liang et al. [25] indicated that *Pseudomonas* is an aerobic bacterium, while *Lactobacillus* is facultatively anaerobic. *Pseudomonas* exhibits superior environmental competitiveness compared to *Lactobacillus*. Marcelli et al. [26] demonstrated that *Pseudomonas* exhibits significantly higher spoilage potential compared to *Lactobacillus* and other bacterial species. Differences in respiratory metabolism further demonstrate the appropriateness of high-barrier packaging materials for preserving the quality of chicken meat.

### 3.2. Effects of Packaging Barrier on TVC in Chilled Chicken During Storage

**Figure 2:**
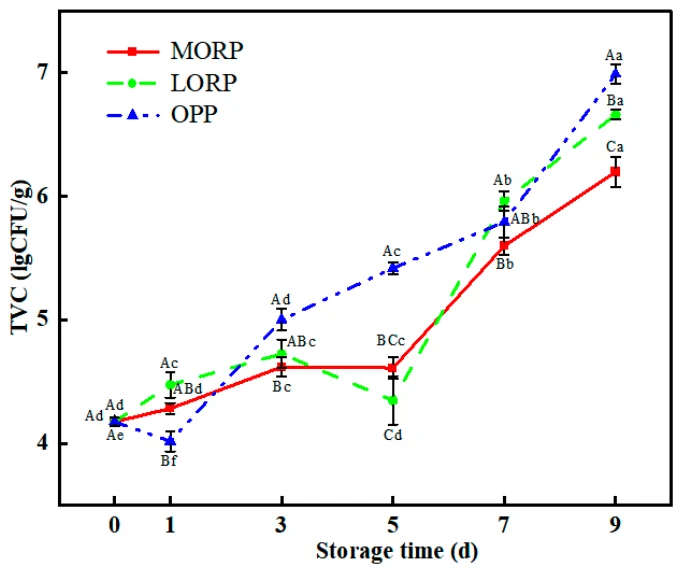
Effect of various barrier packaging on the TVC of chilled chicken stored at 4 °C. For the same treatments, different small letters (a, b, c, d, e, f) indicate a significant difference (*p* < 0.05) in storage time; at the same time point, different capital letters (A, B, C) indicate a significant difference (*p* < 0.05) among the treatments.

Microbial contamination represents a pivotal determinant in the quality deterioration of fresh meat, with particularly pronounced effects during cold chain distribution [27]. Extensive research has established that under refrigerated conditions, *Lactobacillus* and *Pseudomonas* spp. emerge as the predominant spoilage microorganisms [26,27]. These organisms facilitate meat spoilage through the secretion of proteolytic enzymes (e.g., alkaline proteases), siderophores, and exopolysaccharides, which collectively degrade myofibrillar proteins, leading to the production of volatile spoilage metabolites such as ammonia, hydrogen sulfide, and mercaptans [27,28,29]. The initial TVC of chilled chicken meat was 4.17 log CFU/g (Figure 2). All three experimental groups demonstrated a progressive increase in TVC, with statistically significant differences (*p* < 0.05) observed in their growth rates, specifically in the following order: OPP > MORP > LORP. Phase-specific analysis uncovered distinct microbial dynamics: During the initial storage phase (0–3 days), the TVC growth rates in both the MORP and LORP groups were significantly lower (*p* < 0.05) compared to the OPP group, which indicates the enhanced effectiveness of relatively high-barrier packaging materials in inhibiting aerobic bacterial proliferation (Figure 1a,b and Figure 2). Intermediate storage phase (3–5 days): The MORP group displayed near-stagnation in TVC growth, while the LORP group exhibited a distinct downward trend. These observations provide compelling evidence that packaging materials with optimized barrier properties can simultaneously inhibit both aerobic and facultative anaerobic bacterial communities [30]. During days 5–7 of storage, both the MORP and LORP groups exhibited a rapid increase, primarily driven by the exponential growth of facultative anaerobes (*p* < 0.05). The *Pseudomonas* spp. proliferation rate in the OPP group was 7–8 times higher than in the MORP and LORP groups (Figure 1). These differential growth patterns clearly demonstrate that packaging materials with varying oxygen barrier properties exert selective antimicrobial effects on distinct microbial populations. Consequently, matching packaging material permeability to the specific contaminating microbiota’s respiratory characteristics (aerobic vs. facultative anaerobic) represents an effective strategy for optimizing chilled meat preservation. Terminal storage phase (7–9 days): Notably, both the MORP and LORP groups demonstrated a significant reduction (*p* < 0.05) in *Pseudomonas* spp. abundance, likely attributable to organic acid production via *Lactobacillus* metabolism [31] (Kiymaci et al., 2018 [30]). Importantly, the LORP group exhibited a consistent decline in TVC, offering compelling evidence of its efficacy in controlling dominant spoilage microbiota under specified storage conditions.

### 3.3. Effects of Packaging Barrier on pH in Chilled Chicken During Storage

pH serves as a critical biochemical indicator for monitoring fresh meat quality deterioration. As shown in Table 2, chilled chicken exhibited a progressive pH decline throughout storage, revealing ongoing postmortem glycolytic metabolism despite low-temperature conditions [32]. This phenomenon was significantly influenced by microbial community succession and its associated metabolic activities [33]. During the initial storage phase (0–5 days), all packaging groups (OPP, MORP, and LORP) maintained stable pH values (6.70 ± 0.03) without significant intergroup variation (*p* < 0.05). However, by day 7, the OPP group displayed a marked pH reduction to 6.22 ± 0.02 (*p* < 0.05), which correlated strongly (r = 0.89, *p* < 0.01) with *Lactobacillus* spp. dominance (constituting 58.7% ± 3.2% of the total microbiota). LAB proliferation and subsequent lactic acid production were the principal drivers of acidification. *Lactobacillus* was the predominant bacterial genus in the aerated packaging, and it competitively inhibited the growth of *Pseudomonas* by producing organic acids [34]. In contrast, the pH levels in both the MORP and LORP groups exhibited relative stability during this phase. At storage termination (day 9), significant pH reductions were observed in both MORP and LORP, reaching 6.33 ± 0.02 (MORP) and 6.31 ± 0.03 (LORP) (*p* < 0.05 vs. initial values). This progressive acidification correlated strongly with two key factors: depleted oxygen levels in high-barrier packaging, and exponential growth of facultative anaerobes, particularly *Lactobacillus* spp. These physicochemical and microbiological changes were mutually corroborated by the community structure dynamics shown in Figure 1b.

### 3.4. Effects of Packaging Barrier on Color in Chilled Chicken During Storage

Color evolution, a critical organoleptic property governing consumer purchasing decisions, represents a sensitive biochemical marker for real-time freshness evaluation in meat products [35]. Quantitative colorimetric analysis (Table 3) revealed stable L*, a*, and b* values across all treatment groups during initial storage (0–3 days), with no statistically significant intergroup variations (*p* > 0.05). By day 5 of storage, all packaging groups (OPP, MORP, and LORP) showed a pronounced decline in b* values, accompanied by a concurrent but less marked reduction in a* values. This chromatic shift likely may potentially be attributed to the exudation of muscle tissue fluid. With the extended storage period, the a* and b* values of the OPP group exhibited an upward trend, suggesting an increased degree of oxidative deterioration in the chicken meat within this group. This phenomenon was attributed to the inadequate barrier properties of the packaging material and the elevated oxygen concentration inside the packaging [36].

### 3.5. Effects of Packaging Barrier on TVB-N in Chilled Chicken During Storage

**Figure 3:**
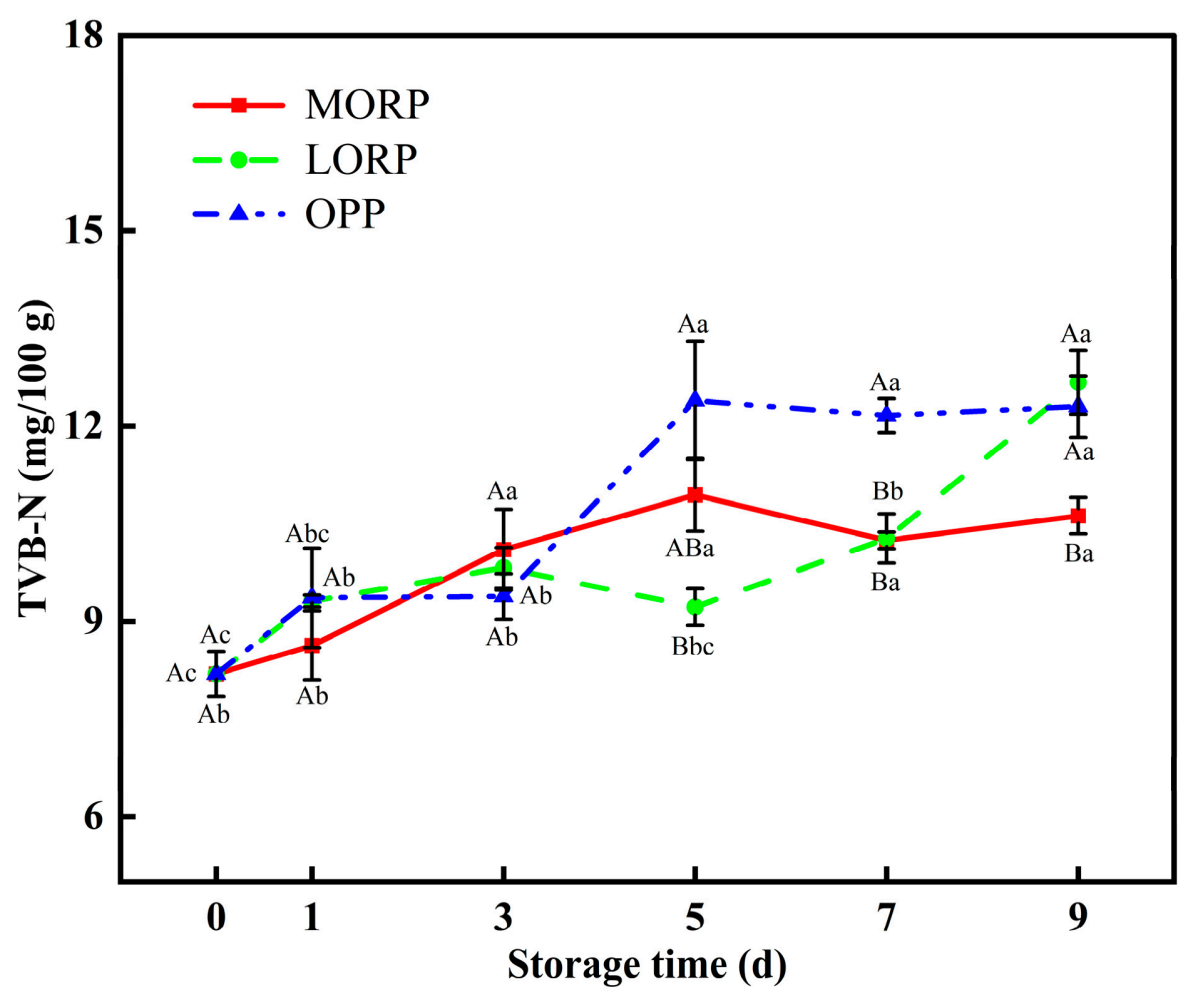
Effect of various barrier packaging on the TVB-N of chilled chicken stored at 4 °C. For the same treatments, different small letters (a, b, c) indicate a significant difference (*p* < 0.05) in storage time; at the same time point, different capital letters (A, B) indicate a significant difference (*p* < 0.05) among the treatments.

Total volatile basic nitrogen (TVB-N) is a well-established biochemical marker for meat freshness evaluation, quantifying the accumulation of alkaline nitrogenous compounds (e.g., ammonia, trimethylamine, and dimethylamine) derived from microbial and endogenous enzymatic proteolysis [37]. As shown in Figure 3, the baseline TVB-N content of fresh chicken samples was determined to be 8.19 ± 0.23 mg/100 g, which falls well within the acceptable range for premium-quality poultry (<10 mg/100 g) according to EU Regulation 2073/2005 and Fresh (frozen) livestock and poultry products (15 mg/100 g) according to GB 2707-2016 [38]. The TVB-N values of the OPP, MORP, and LORP exhibited distinct growth trends during storage. During the early storage phase (0–3 days), the MORP and LORP groups exhibited marginally higher TVB-N values (10.10 ± 0.62 and 9.82 ± 0.31 mg/100 g at day 3, respectively) compared to the OPP group (9.38 ± 0.35 mg/100 g) (*p* < 0.05). This initial difference correlated strongly with the relative abundance of proteolytic bacteria. With the extension of storage time, variations in oxygen levels within the packaging result in substantial alterations to the microbial community structure. The abundance of *Pseudomonas* in the OPP group was significantly higher compared to that in the MORP and LORP groups, and the TVB-N values exhibited a more rapid increasing trend. This suggests that *Pseudomonas* plays a predominant role in protein degradation and the formation of ammonia and amine decomposition products [21]. TVB-N values differed significantly among groups, measuring 10.94 (OPP), 9.22 (MORP), and 12.39 mg/100 g (LORP) on day 5, with MORP (10.24 mg/100 g) and LORP (10.27 mg/100 g) showing significantly lower values than OPP (12.16 mg/100 g) by day 7 (*p* < 0.05). After 9 days of storage, TVB-N levels followed the order: OPP > LORP > MORP. The TVB-N level of the OPP showed a strong correlation with *Pseudomonas* abundance across groups. The MORP controls *Pseudomonas* spp. proliferation, thereby retarding TVB-N accumulation and preserving chilled chicken freshness.

### 3.6. Effects of Packaging Barrier on Sulfhydryl Level in Chilled Chicken During Storage

**Figure 4:**
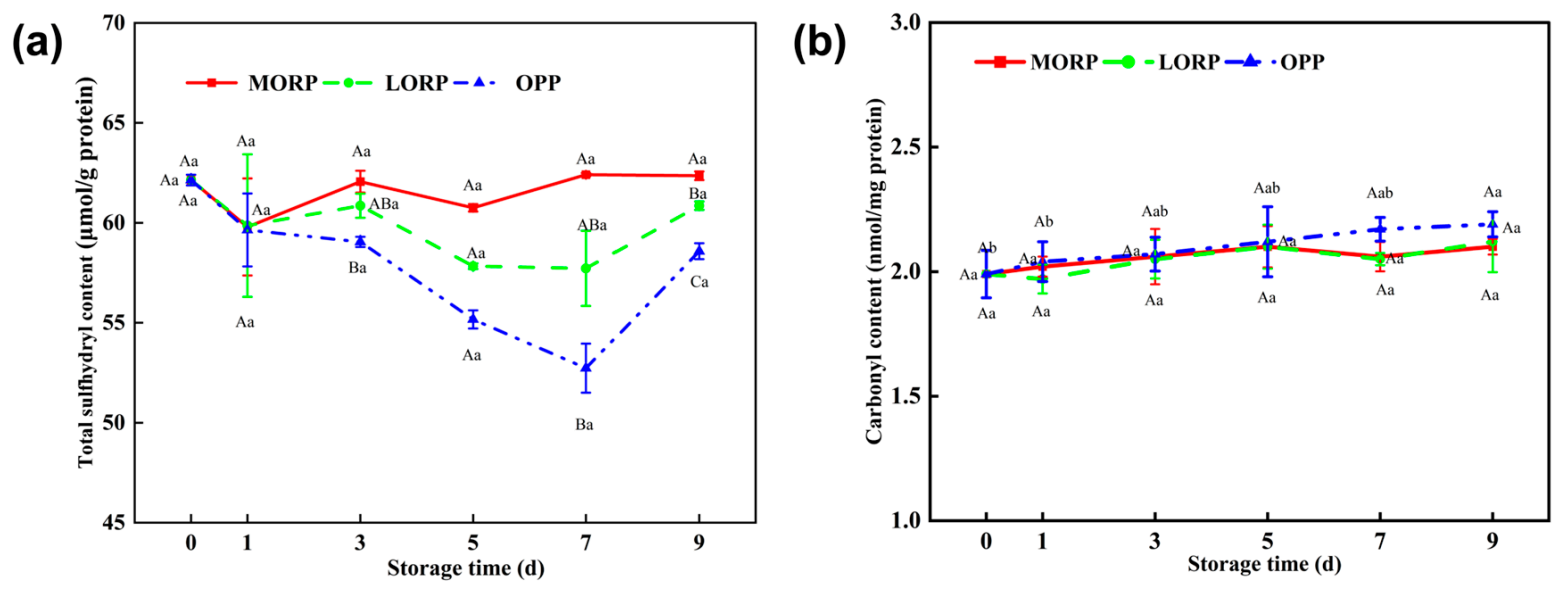
Effect of various barrier packaging on the total sulfhydryl (**a**) and carbonyl content (**b**) of chilled chicken stored at 4 °C. For the same treatments, different small letters (a, b) indicate a significant difference (*p* < 0.05) in storage time; at the same time point, different capital letters (A, B, C) indicate a significant difference (*p* < 0.05) among the treatments.

The sulfhydryl group (-SH), a reactive functional group in protein molecules, serves as a key biochemical indicator for assessing protein oxidation levels [39]. This study systematically characterized the temporal variations in protein thiol content of chilled chicken meat across different packaging regimes during refrigerated storage, and the results are shown in Figure 4. At the initial stage, the sulfhydryl content in fresh chicken meat was determined to be 62.14 µmol/g protein. Protein sulfhydryl content in the MORP group demonstrated stability throughout storage (62.14 ± 0.27 to 62.35 ± 0.22 μmol/g protein). In contrast, both OPP and LORP groups exhibited biphasic thiol kinetics: an initial decline (OPP: 62.14 → 52.73 ± 1.22; LORP: 62.14 → 57.72 ± 1.88 μmol/g protein) followed by partial recovery (day 9: OPP 58.58 ± 0.40; LORP 60.86 ± 0.21 μmol/g protein). The oxidative phase was significantly more pronounced in OPP versus LORP (Δsulfhydryl = 3.56 vs. 1.27 μmol/g protein, *p* < 0.05). This observation is consistent with the findings of Bekhit et al. [28], demonstrating a strong inverse correlation between thiol group depletion and protein oxidation progression. During the storage period of 3 to 7 days, the sulfhydryl content in each group exhibited a significant decrease, suggesting that this phase is a critical period for protein oxidation. By day 7, the sulfhydryl content in both the OPP and LORP groups had decreased to their respective lowest values of 52.73 and 57.72 µmol/g protein. During the late storage phase (7–9 days), a significant recovery in sulfhydryl content was observed (ΔSH = +2.92 and +1.55 μmol/g/day for OPP and LORP, respectively, *p* < 0.05), likely due to redox equilibrium establishment and subsequent reduction reactions [40]. Across the entire storage period, sulfhydryl retention consistently adhered to the hierarchy MORP > LORP > OPP, unequivocally demonstrating the superior efficacy of high-barrier packaging in mitigating protein oxidation.

### 3.7. Effects of Packaging Barrier on Carbonyl Level in Chilled Chicken During Storage

Protein carbonyls serve as crucial biomarkers for oxidative modification, with their concentration directly reflecting the degree of protein oxidation [41]. Oxidative stress induces carbonylation of amino acid residues in protein molecules, generating carbonyl compounds that modify protein structure and function, ultimately affecting meat quality [42]. As shown in Figure 4b, all experimental groups exhibited a gradual increase in carbonyl content during storage, though the overall magnitude of this increase remained moderate. Notably, the three groups showed comparable carbonyl levels during the initial storage period (0–5 days). However, significant differences emerged in the later phase (5–9 days), with the OPP group demonstrating substantially higher carbonyl content (*p* < 0.05) compared to both the LORP and MORP groups. Furthermore, while the OPP group displayed a continuous upward trend in carbonyl formation, the other two groups maintained relatively stable levels throughout the storage period. These results provide compelling evidence that LORP and MORP materials combined with low-temperature storage conditions more effectively maintain protein stability in chilled chicken meat by mitigating oxidative damage.

### 3.8. Effects of Packaging Barrier on Volatile Substances in Chilled Chicken During Storage

**Figure 5:**
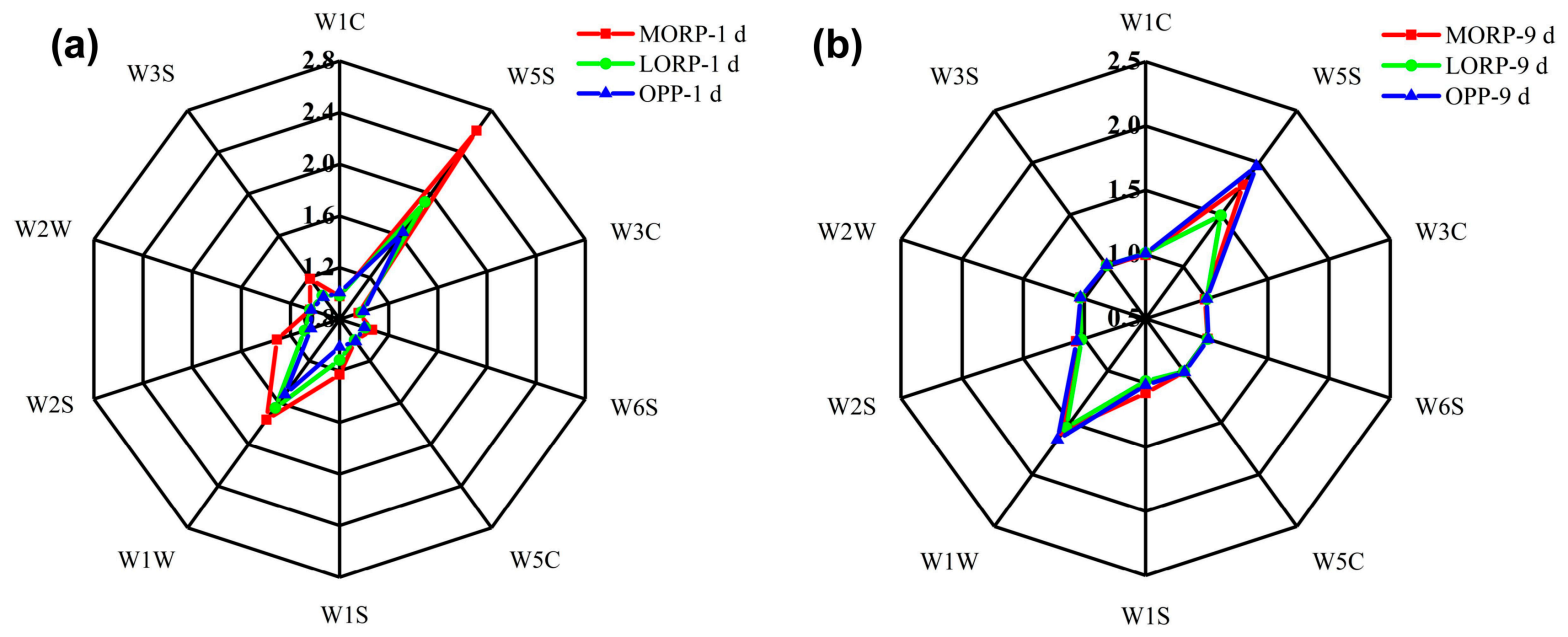
Effect of various barrier packaging on the volatile odor of chilled chicken stored at 4 °C at 1 d (**a**) and 9 d (**b**).

Proteins and lipids serve as the principal chemical constituents of chilled chicken meat. During refrigerated storage, oxidative degradation of these components generates characteristic spoilage volatiles, including ammonia, amines, organic acids, and sulfur-containing compounds. Electronic nose analysis was employed to monitor dynamic changes in volatile organic compounds (VOCs) across different packaging treatment groups, and the results are shown in Figure 5. Among the sensor array, the W5S (nitrogen oxide-sensitive) and W1W (sulfide-sensitive) sensors displayed the most pronounced response signals throughout the storage period. Temporal analysis revealed that in the OPP-packaged group, both sensors exhibited progressively increasing response values, with W5S demonstrating significantly greater signal intensity than W1W (*p* < 0.05). This differential response pattern indicates that nitrogenous compounds (particularly ammonia and volatile amines) constitute the predominant spoilage indicators in chicken meat, a conclusion that corroborates previous findings [28]. Comparative analysis of sensor responses among treatment groups (Figure 5b) established a clear hierarchy: OPP > MORP > LORP. This gradation in signal intensity provides compelling evidence that both MORP and LORP systems exhibit superior efficacy in suppressing the generation of key spoilage metabolites compared to conventional OPP.

### 3.9. Effects of Packaging Barrier on the Microstructure of Muscle Fibers in Chilled Chicken During Storage

**Figure 6:**
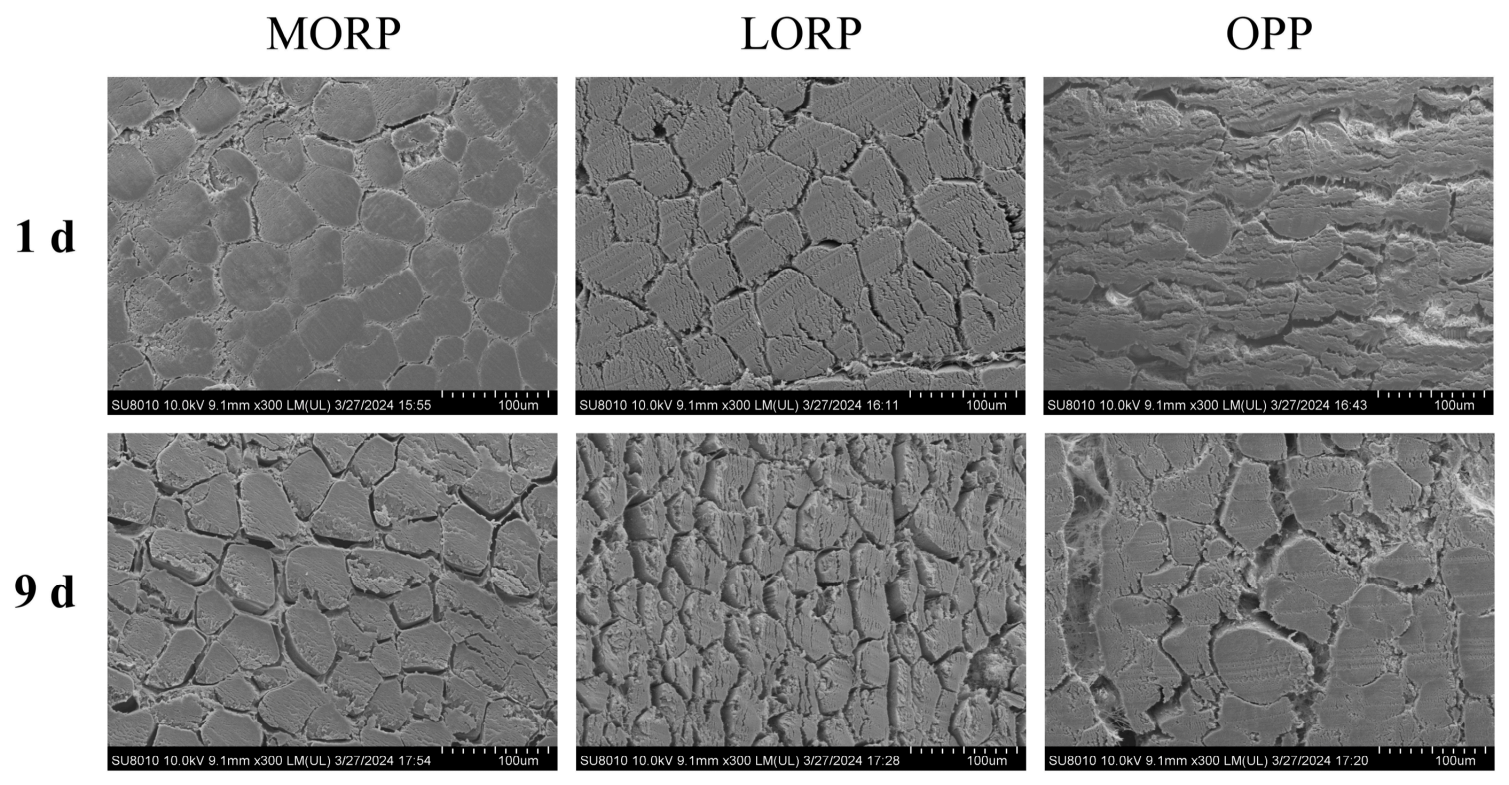
Effect of various barrier packaging on the microstructure of chilled chicken stored at 4 °C at 1 and 9 d.

The differential effects of oxygen-resistant packaging materials on the microstructural integrity of chilled chicken meat and the results are shown in Figure 6. Following one day of storage, the MORP-packaged samples preserved ultrastructural organization closely resembling their original state, whereas the LORP group demonstrated early signs of myofibrillar separation. Distinct microstructural alterations emerged across all treatment groups following progressive storage through day 9. The MORP samples showed moderate myofibrillar contraction with developing inter-fibrillar gaps. More pronounced structural disorganization was evident in LORP specimens, manifesting as expanded intermyofibrillar spaces. The most severe degradation occurred in OPP-packaged samples, displaying extensive myofibrillar contraction and architectural disruption. SEM analysis demonstrated that prolonged storage led to gradual expansion of intermyofibrillar spaces and progressive deterioration of tissue integrity in chicken muscle. Notably, at equivalent storage durations, samples from the MORP group (with the lowest oxygen transmission rate, OTR) consistently maintained a denser myofibrillar arrangement and smoother cross-sectional morphology compared to other groups. This structural preservation likely stems from the suppressive effect of reduced oxygen permeation on protein oxidation, which in turn enhances the microstructural stability of the muscle tissue.

## 4. Conclusions

This study selected three packaging materials with different oxygen barrier properties [23.95, 1631.44, and 10,600.00 cm^3^/(m^2^·24 h·0.1 MPa)] to systematically evaluate their effects on the freshness preservation of vacuum-packaged chilled chicken, aiming to determine the appropriate barrier requirements. Medium–low barrier materials (LORP and MORP) inhibited the proliferation of dominant spoilage bacteria *Pseudomonas* spp. and significantly reduced the total bacterial growth rate. Meanwhile, these two materials exhibited antioxidant effects, increasing sulfhydryl content by 0.25–18.35% and decreasing carbonyl content by 0.48–5.07%. Regarding quality maintenance, LORP and MORP materials delayed the formation of spoilage markers, including nitrogen oxides and sulfides, while maintaining good textural properties of chicken meat by preserving the integrity of myofibrillar structures. Packaging materials with medium–low oxygen transmission rates (23.95–1631.44 cm^3^/(m^2^·24 h·0.1 MPa)) are more suitable for chilled chicken preservation.

## Figures and Tables

**Table 2:** Effect of various barrier materials on the pH value of chilled chicken stored at 4 °C.

Storage Time (d)		MORP	LORP	OPP
pH value	0	7.25 ± 0.02 ^Aa^	7.25 ± 0.02 ^Aa^	7.25 ± 0.02 ^Aa^
	1	7.25 ± 0.01 ^Aa^	7.25 ± 0.01 ^Aa^	7.27 ± 0.01 ^Aa^
	3	7.25 ± 0.04 ^Aa^	7.25 ± 0.01 ^Aa^	7.25 ± 0.01 ^Aa^
	5	7.25 ± 0.04 ^Aa^	7.23 ± 0.01 ^Aa^	7.24 ± 0.02 ^Aa^
	7	7.22 ± 0.03 ^Aa^	7.22 ± 0.01 ^Aa^	6.26 ± 0.34 ^Bb^
	9	6.33 ± 0.04 ^Ab^	6.31 ± 0.01 ^Ab^	6.37 ± 0.03 ^Ab^

Values represent means ± SE (*n* = 6). For the same treatments, different small letters (a, b) indicate a significant difference (*p* < 0.05) in storage time; at the same time point, different capital letters (A, B) indicate a significant difference (*p* < 0.05) among the treatments. Abbreviations: MORP: stored at 4 °C and treated with medium-oxygen-resistant packaging; LORP: stored at 4 °C and treated with low-oxygen-resistant packaging; OPP: stored at 4 °C and treated with oxygen-permeable packaging.

**Table 3:** Effect of various barrier materials on the color of chilled chicken stored at 4 °C.

Storage Time (d)		MORP	LORP	OPP
L*	0	62.14 ± 1.15 ^Ab^	62.14 ± 1.15 ^Ab^	62.14 ± 1.15 ^Ab^
	1	70.17 ± 0.71 ^Aa^	70.86 ± 1.41 ^Aa^	69.21 ± 0.84 ^Aa^
	3	72.30 ± 0.68 ^Aa^	72.60 ± 0.59 ^Aa^	71.16 ± 0.53 ^Aa^
	5	60.20 ± 0.51 ^Abc^	59.84 ± 0.85 ^Ab^	57.63 ± 0.48 ^Bc^
	7	58.39 ± 0.84 ^Ac^	56.71 ± 0.74 ^Ac^	57.15 ± 1.59 ^Ac^
	9	58.75 ± 0.96 ^Ac^	59.37 ± 0.48 ^Abc^	58.67 ± 0.83 ^Ac^
a*	0	0.95 ± 0.56 ^Ab^	0.95 ± 0.56 ^Ac^	0.95 ± 0.56 ^Ac^
	1	0.87 ± 0.26 ^Ab^	0.55 ± 0.10 ^Ac^	0.78 ± 0.14 ^Ac^
	3	1.13 ± 0.18 ^Bb^	1.64 ± 0.30 ^Aa^	1.45 ± 0.23 ^Aab^
	5	1.40 ± 0.19 ^Aa^	1.39 ± 0.13 ^Aabc^	1.29 ± 0.22 ^Aabc^
	7	1.34 ± 0.23 ^Aab^	1.49 ± 0.26 ^Aab^	1.46 ± 0.19 ^Aab^
	9	1.57 ± 0.23 ^Aa^	1.48 ± 0.22 ^Aab^	1.79 ± 0.16 ^Aa^
b*	0	7.62 ± 0.85 ^Ac^	7.62 ± 0.85 ^Abc^	7.62 ± 0.85 ^Ac^
	1	13.53 ± 0.39 ^Aa^	12.83 ± 0.43 ^Aa^	13.33 ± 0.59 ^Aa^
	3	10.58 ± 0.47 ^Cb^	12.15 ± 0.63 ^Ba^	14.57 ± 0.58 ^Aa^
	5	7.50 ± 0.68 ^Ac^	6.40 ± 0.63 ^Acd^	7.63 ± 0.3 ^Ac^
	7	10.51 ± 0.50 ^Ab^	9.16 ± 0.50 ^ABb^	9.01 ± 0.57 ^ABbc^
	9	6.00 ± 0.63 ^Bc^	5.74 ± 0.43 ^Bd^	9.58 ± 0.62 ^Ab^
∆E*	1	9.97	10.17	9.09
	3	10.58	11.42	11.40
	5	2.00	2.64	4.53
	7	4.75	5.67	5.21
	9	3.81	3.39	4.07

Values represent means ± SE (*n* = 6). For the same treatments, different small letters (a, b, c, d) indicate a significant difference (*p* < 0.05) in storage time; at the same time point, different capital letters (A, B, C) indicate a significant difference (*p* < 0.05) among the treatments. Abbreviations: MORP: stored at 4 °C and treated with medium-oxygen-resistant packaging; LORP: stored at 4 °C and treated with low-oxygen-resistant packaging; OPP: stored at 4 °C and treated with oxygen-permeable packaging.

## Data Availability

The original contributions presented in the study are included in the article. Further inquiries can be directed to the corresponding author.
